# Digital Governance at the Street Level: A Mixed-Methods Study of Systemic Resilience and ‘Human-in-the-Loop’ Telemedicine in Rural Thailand

**DOI:** 10.3390/ijerph23040490

**Published:** 2026-04-13

**Authors:** Nathachon Tarnthong, Chitralada Chaiya

**Affiliations:** College of Politics and Governance, Mahasarakham University, Kantharawichai District, Mahasarakham 44150, Thailand

**Keywords:** telemedicine, home ward, mixed methods, human-in-the-loop, service co-production, social sustainability, rural healthcare, Thailand

## Abstract

**Highlights:**

**Public health relevance—How does this work relate to a public health issue?**

- **Digital Divide in Rural Care:** Highlights how standardized digital health often fails in resource-constrained settings, exacerbating inequities for underserved rural populations.
- **Sustainability of Telemedicine in LMICs:** Identifies a critical research gap in transitioning telemedicine from an experimental niche to a sustainable pillar of Universal Health Coverage (UHC).

**Public health significance—Why is this work of significance to public health?**

- **Pragmatic Utility as a Driver:** Demonstrates that high patient satisfaction is catalyzed by economic and logistical relief—mitigating the “medical poverty trap”—rather than technological sophistication.
- **Socio-Technical Resilience:** Validates the “Human-in-the-Loop” model where Village Health Volunteers (VHVs) serve as essential “digital bridges” to compensate for infrastructural deficiencies.

**Public health implications—What are the key implications or messages for practitioners, policy makers and/or researchers in public health?**

- **Decentralized Digital Governance:** Advocates for a shift toward governance frameworks that formally recognize and compensate the discretionary efforts of street-level healthcare workers.
- **Mitigating the Resilience–Exploitation Nexus:** Proposes institutionalizing support for “invisible labor” to prevent provider burnout and ensure the long-term durability of co-produced health models.

**Abstract:**

While telemedicine has proliferated globally, its sustainable implementation in resource-constrained settings remains understudied. This study evaluates the efficacy, determinants of patient satisfaction, and systemic resilience of a “Home Ward” model at a rural Thai community hospital. Employing a convergent mixed-methods design, we surveyed 51 participants and conducted in-depth interviews with service users (*n* = 5) and a multidisciplinary team (*n* = 7). Multiple linear regression revealed high patient satisfaction ($\bar{x}$ = 3.70), explaining 67.3% of the variance (R^2^ = 0.673). Notably, Perceived Usefulness (β = 0.589, *p* < 0.001) and the Effectiveness of Symptom Monitoring (β = 0.317, *p* < 0.05) significantly predicted satisfaction. Conversely, Overall System Quality was not a significant predictor (β = 0.142, *p* > 0.05), highlighting a ‘Low-Tech, High-Touch’ paradox. Qualitative analysis elucidated this through the “Human-in-the-Loop” mechanism, where Village Health Volunteers (VHVs) and healthcare providers bridge the digital divide. However, the study identifies an “invisible workload”—non-formalized discretionary effort—that sustains this resilience. Findings suggest that rural digital health governance should prioritize human intermediaries and pragmatic utility over purely technical upgrades. The study concludes that long-term sustainability requires institutionalizing human support networks while mitigating the exploitation of healthcare personnel’s goodwill.

## 1. Introduction

The proliferation of digital health technologies has fundamentally reshaped the landscape of global healthcare delivery. Accelerated by the COVID-19 pandemic, telemedicine has transitioned from a niche innovation to a critical component of universal health coverage, offering a viable solution to overcome healthcare delivery challenges [1]. In the post-pandemic era, models such as “Hospital-at-Home” or “Home Ward” have gained significant traction as strategies to decongest hospitals and provide patient-centered care [2]. However, the global discourse on telemedicine is often characterized by a “technological determinism” bias, predominantly driven by evidence from high-income countries with robust digital infrastructure [3].

This narrative frequently overlooks the complex realities of Low- and Middle-Income Countries (LMICs), particularly in rural settings where the “digital divide,” resource scarcity, and workforce shortages pose formidable barriers to adoption [4]. In these resource-limited settings, the lack of infrastructure and training often hinders the successful integration of eHealth systems [4]. Consequently, there remains a critical knowledge gap regarding how telemedicine models can be sustainably implemented in such environments without relying heavily on high-end technology.

Recent scholarship suggests that in LMICs, the success of digital health interventions depends less on technological sophistication and more on the adaptability of the system to local contexts. The Technology Acceptance Model (TAM) posits that for users in these settings, “perceived usefulness”—the pragmatic ability of technology to solve livelihood problems—outweighs its ease of use or advanced features [5,6]. Furthermore, the sustainability of these systems often relies on social capital [6] and service co-production [7], where community health workers and families act as intermediaries to bridge the technological gap. The concept of “Human-in-the-Loop” is particularly relevant here, emphasizing that human elements and therapeutic alliances remain central to effective healthcare delivery, even in digital formats [8].

Despite these theoretical advancements, current literature on digital health governance exhibits four critical gaps. First, a contextual gap exists, as most telemedicine models are validated in urban, high-resource settings, leaving rural LMIC realities underexplored. Second, there is a technological gap concerning how systems function when ‘high-end’ infrastructure is absent. Third, a human-element gap remains regarding how social capital and human mediation functionally compensate for these technical deficiencies. Finally, a methodological gap persists in the lack of mixed-methods evidence that triangulates quantitative adoption metrics with qualitative ‘street-level’ perspectives. This study addresses these voids by investigating the ‘Home Ward’ model in rural Thailand.

Thailand offers a unique context to explore this dynamic. Despite having a successful Universal Health Coverage (UHC) scheme, its rural healthcare system still faces significant disparities. To address this, the Thai Ministry of Public Health has promoted the “Home Ward” policy to decentralize care. This study focuses on Rongkham Hospital, a rural community hospital in Kalasin Province, which has implemented a telemedicine-based Home Ward system to manage the influx of patients amid resource constraints.

To analyze the sustainability of such interventions, this study operationalizes two critical concepts. First, ‘Systemic Resilience’ is defined as the adaptive capacity of a healthcare system to maintain clinical functions and service continuity amidst structural resource constraints through socio-technical workarounds. Second, the study examines the ‘Invisible Workload,’ conceptualized within the framework of Street-Level Bureaucracy (SLB). This refers to the non-formalized, discretionary efforts and emotional labor performed by frontline health workers—such as after-hours digital communication and informal patient monitoring—that are essential for system survival but are often unrecognized in formal policy metrics.

To provide a comprehensive analysis, this study employs a dual-theoretical framework tailored to its convergent mixed-methods design. The Technology Acceptance Model (TAM) serves as the primary lens for the quantitative strand, allowing for the systematic identification of adoption determinants—specifically how perceived usefulness and system quality influence patient satisfaction. Conversely, the ‘Human-in-the-Loop’ (HITL) framework guides the qualitative inquiry. HITL is utilized to explore the compensatory processes where human agency, represented by healthcare staff and Village Health Volunteers (VHVs), mitigates technological gaps. By synthesizing these frameworks, the study moves beyond mere description to explain how social capital and human mediation functionally sustain digital health governance in resource-constrained environments.

This research aims to evaluate the efficacy, determinants of patient satisfaction, and systemic resilience of the Home Ward model in a rural Thai setting. By employing a Convergent Mixed-Methods Design [9], the study investigates how system quality, monitoring processes, and perceived usefulness influence patient satisfaction, and how healthcare providers and community networks co-produce services to overcome technological limitations.

To address these gaps, this research is guided by three specific Research Questions:

- RQ1: To what extent do digital system quality and monitoring processes influence patient satisfaction in a resource-constrained rural context?
- RQ2: How does perceived usefulness mediate the relationship between technological infrastructure and system adoption?
- RQ3: How do human intermediaries (VHVs and MDTs) co-produce ‘Systemic Resilience’ to overcome structural and technological limitations? By answering these questions, the study proposes a ‘Low-Tech, High-Touch’ model

This study contributes to the global literature by proposing a “Low-Tech, High-Touch” model for sustainable digital health governance, challenging the high-tech paradigm and offering a blueprint for other developing nations.

## 2. Literature Review

### 2.1. Theoretical Frameworks for Telemedicine Adoption in Resource-Limited Settings

The adoption of telemedicine and eHealth systems, particularly within the context of Low- and Middle-Income Countries (LMICs), requires a robust theoretical underpinning to elucidate the complex interplay between technology and user behavior. The Technology Acceptance Model (TAM) and the Unified Theory of Acceptance and Use of Technology (UTAUT) serve as pivotal frameworks for this purpose.

Originally developed by Davis in 1989, TAM posits that perceived usefulness and perceived ease of use are the primary determinants of technology acceptance. These constructs remain critically relevant when evaluating eHealth systems in resource-limited settings, where the practical utility of technology often outweighs its complexity [4,6]. The relevance of these models has gained significant traction in LMICs, driven by the urgent need to overcome healthcare delivery challenges exacerbated by the COVID-19 pandemic [1].

Empirical studies utilizing these frameworks have consistently demonstrated that individual factors, such as self-efficacy and effort expectancy, along with social influence, significantly impact the attitudes and behavioral intentions of healthcare professionals toward telemedicine [10]. The UTAUT model, which extends TAM by incorporating constructs like social influence and facilitating conditions, has been effectively applied to assess telemedicine acceptance among providers. Research indicates that performance expectancy—analogous to perceived usefulness—and effort expectancy are critical predictors of behavioral intention [11]. For instance, a study employing an extended UTAUT model in Ethiopia highlighted that self-efficacy and effort expectancy significantly influenced providers’ attitudes, underscoring the applicability of these constructs in LMIC contexts [1].

Furthermore, the integration of mobile health (mHealth) services, a subset of telemedicine, has been identified as a preferred modality in these regions, offering a promising avenue for enhancing healthcare delivery where traditional infrastructure is lacking [12]. To address the specific constraints of rural and resource-limited environments, modified TAM and UTAUT models have been adapted to include context-specific factors such as perceived risk and technical infrastructure [13]. These adaptations are crucial for understanding adoption barriers in settings where infrastructure instability and lack of training are prevalent. Overall, these frameworks provide a robust theoretical lens for understanding the adoption of telemedicine in LMICs, emphasizing the need for targeted interventions that address both the technological and contextual facilitators of acceptance.

### 2.2. The Donabedian Model: A Framework for Quality Assessment in Digital Health

The Donabedian Model [14] remains a seminal framework in healthcare quality assessment, positing that quality of care is a linear function of three interrelated components: structure, process, and outcome. This model provides a comprehensive lens for systematically linking structural resources and care delivery processes to tangible health outcomes [15]. Its adaptability has been demonstrated across diverse domains, ranging from clinical care to digital health policy and education.

In the context of Structure, which encompasses the attributes of the setting in which care occurs, recent adaptations of the model have focused on governance and infrastructure. Saheb and Saheb (2024) utilized the model to categorize national digital health strategies, identifying governance frameworks as critical structural elements [16]. Similarly, in the integration of eHealth systems, the alignment of technology with human resources is identified as a fundamental structural indicator necessary for successful implementation [17]. This structural foundation extends to staffing and equipment availability, which Ghofrani (2024) noted as key determinants of institutional performance in nursing education settings [18].

The Process component, referring to what is actually done in giving and receiving care, serves as the bridge between resources and results. Research in diabetes care elucidates how process variables, particularly the utilization of human resources and communication, significantly influence the delivery of service [19]. Furthermore, in vulnerable settings such as nursing homes, process measures like medication management are critical links that connect structural factors (e.g., staffing levels) to patient safety outcomes [20].

Ultimately, these antecedents culminate in Outcome measures, which reflect the effects of care on the health status of patients and populations. The model’s application in surgical tourism underscores the importance of optimizing structure and process dimensions to ensure patient safety and service quality [21]. Crucially, empirical evidence suggests that robust structural and process variables are significant predictors of service satisfaction and quality of life [19]. This highlights the model’s utility in evaluating complex healthcare interventions, such as Home Ward systems, where the interplay between technological readiness (structure) and continuous monitoring (process) directly impacts patient satisfaction (outcome).

### 2.3. Human-in-the-Loop and Relationship-Based Care in Telehealth

While technological infrastructure provides the mechanism for telehealth, the concept of “Human-in-the-Loop” posits that human elements remain central to effective healthcare delivery. In the digital realm, relationship-based care—particularly the therapeutic alliance—is crucial for enhancing patient engagement and clinical outcomes. The Therapeutic Relational Connection (TRC) in telehealth is defined as a mutually responsive dynamic between patient and provider, grounded in respect, understanding, and cultural humility, which is essential for achieving therapeutic aims [10].

The efficacy of this alliance in digital care relies heavily on trust, the perception of interactions, and a sense of being considered, all of which are critical predictors of patient adherence [22]. Contrary to concerns that technology creates distance, evidence indicates that the therapeutic alliance in videoconferencing psychotherapy (VCP) is comparable to in-person interactions, suggesting that digital formats can indeed maintain effective therapeutic relationships when managed correctly [23].

However, in rural and remote communities, the application of telehealth requires more than just connectivity; it demands culturally informed approaches to build trust and ensure privacy [24,25]. This is particularly significant for specific populations, such as Indigenous or First Nations peoples, where cultural safety, spiritual identity, and community engagement are vital prerequisites for effective telehealth adoption [26].

In this context, the role of community intermediaries becomes paramount. The literature implies that Village Health Volunteers (VHVs) or local health workers act as “digital bridges” in community-driven initiatives. By integrating local knowledge and support systems, these human intermediaries enhance the therapeutic alliance, ensuring that telehealth initiatives are culturally relevant and accessible [27]. Consequently, integrating human elements and cultural considerations into telehealth design significantly improves patient-centered outcomes across diverse populations.

The role of human intermediaries in digital health transcends mere technical assistance; it represents a form of ‘Compensatory Systemic Resilience.’ Unlike purely technical resilience, this mechanism relies on the discretionary power of street-level bureaucrats [28]. In the context of rural Thailand, this study defines the ‘Invisible Workload’ as a manifestation of this discretion, where Village Health Volunteers (VHVs) and hospital staff absorb the ‘digital friction’ of the system. By defining these terms through an SLB lens, we can move beyond fashionable terminology to evaluate how personal altruism and extra-role behaviors [29] act as the primary engines of telemedicine in resource-constrained environments.

### 2.4. Co-Production and Social Capital in Rural Healthcare Sustainability

The sustainability of healthcare services in rural environments is increasingly contingent upon the concept of service co-production, which posits that positive health outcomes result from the active, reciprocal collaboration between healthcare providers and patients. This paradigm acknowledges the distinct nature of services as opposed to products, emphasizing the interdependent roles of all stakeholders in the delivery process [9].

In rural settings, where workforce shortages and geographical barriers frequently jeopardize service continuity, co-production becomes a strategic necessity rather than a mere option. Integrated approaches that leverage social capital are essential to enhance service delivery and workforce retention [30]. Social capital, defined as the networks and norms that facilitate collective action, functions as a critical resource in rural healthcare by fostering trust and collaboration among community members and between communities and providers [6].

Crucially, intermediaries—such as nonprofit organizations or community health workers—significantly contribute to this process by building trust and facilitating communication between government entities and rural residents, thereby catalyzing co-production efforts [31]. In multicultural and diverse rural contexts, successful co-production demands not only structural collaboration but also a deep understanding of cultural nuances to ensure effective service delivery [32]. Evidence from regions like the Haor communities in Bangladesh demonstrates that establishing trust networks and organizing community participation can significantly improve healthcare outcomes [33].

Ultimately, the integration of social capital into co-production models is vital for achieving sustainable and equitable healthcare services in rural areas. By enhancing community resilience and facilitating the development of effective partnerships, this approach ensures that the health system remains robust even in resource-constrained environments [34,35].

### 2.5. Research Gaps

Despite the proliferating literature on telemedicine and Hospital-at-Home (HaH) models, significant lacunae remain when translating these concepts to resource-constrained settings. A critical review reveals four primary research gaps that this study aims to address:

1. The Contextual Gap (Developed vs. Developing Nations): Most empirical evidence on HaH efficacy stems from high-income countries with advanced digital infrastructure. There is a paucity of research focusing on Low- and Middle-Income Countries (LMICs), particularly in rural community hospitals where resources are scarce. It remains unclear whether models successful in the West can be replicated in rural Thailand without significant infrastructure investment.

2. The Technological Gap (High-Tech vs. Appropriate Technology): Current literature often conflates telemedicine success with cutting-edge technologies. However, the Technology Acceptance Model (TAM) suggests that in rural contexts, pragmatic utility outweighs technological sophistication. Limited research explores the efficacy of “Appropriate Technology”—such as basic mobile applications (e.g., LINE) combined with simple medical devices—in managing inpatient-level care.

3. The Human-Element Gap (Tech-Centric vs. Relationship-Based Care): While many studies focus on technical feasibility, fewer examine the “Human-in-the-Loop” aspect, specifically the role of intermediaries like Village Health Volunteers (VHVs). The mechanism of Service Co-production—how community members compensate for technological limitations in rural settings—has not been sufficiently explored.

4. The Methodological Gap (Unidimensional vs. Mixed-Methods): Previous studies often rely solely on quantitative metrics or qualitative insights in isolation. Few utilize a Convergent Mixed-Methods Design to juxtapose high patient satisfaction against the operational resilience required from healthcare providers. This study bridges this gap by integrating quantitative data with qualitative insights into systemic sustainability.

### 2.6. Towards an Integrated Conceptual Framework

**Figure 1:**
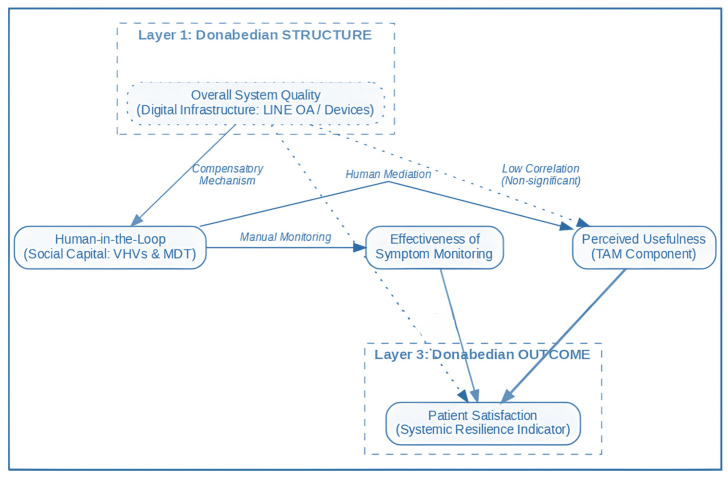
Integrated Conceptual Model of Systemic Resilience in Rural Telemedicine.

To address the theoretical fragmentation often found in digital health studies, this research proposes an Integrated Conceptual Model (Figure 1) that synthesizes three distinct theoretical pillars. First, Donabedian’s (1966) Structure-Process-Outcome (SPO) framework provides the overarching institutional logic [14]. In this study, ‘Structure’ is operationalized through System Quality (the digital infrastructure), while ‘Process’ is represented by the Human-in-the-Loop mechanism and Social Capital (VHVs and MDT interactions).

Second, these institutional components are mapped onto the Technology Acceptance Model (TAM) constructs. We hypothesize that the structural limitations of a rural hospital (Structure) are mediated by the ‘Human-in-the-Loop’ process, which directly influences the user’s Perceived Usefulness (Process) and ultimately leads to Patient Satisfaction (Outcome). This integrated approach allows for an analysis of how human social capital compensates for technical structural deficiencies—a phenomenon we term ‘Compensatory Systemic Resilience’. By linking Donabedian’s processual lens with TAM’s cognitive variables, the model explains why high satisfaction can persist even when technical system quality is suboptimal.

Figure 1 illustrates the study’s integrated conceptual framework, which synthesizes Donabedian’s Structure-Process-Outcome (SPO) model with the Technology Acceptance Model (TAM) and the Human-in-the-Loop (HITL) mechanism. The framework is categorized into three analytical layers:

1. Donabedian STRUCTURE (Layer 1): This layer represents the foundational digital infrastructure, operationalized as Overall System Quality. In this rural context, it refers to the technical reliability of the LINE application and remote monitoring devices. The dotted line to outcomes indicates the hypothesized ‘Low-Path,’ where technical quality alone may not directly drive satisfaction in resource-constrained settings.
2. Donabedian PROCESS (Layer 2): This core mediating layer represents the socio-technical adaptation of the system. It consists of the Human-in-the-Loop (HITL) mechanism—where Village Health Volunteers (VHVs) and multidisciplinary teams act as a ‘digital bridge’—which in turn enhances Perceived Usefulness and the Effectiveness of Symptom Monitoring. This layer explains how social capital compensates for structural limitations.
3. Donabedian OUTCOME (Layer 3): The final stage is Patient Satisfaction, which serves as an empirical indicator of Systemic Resilience.

The model highlights two critical pathways: the Compensatory Mechanism (the shift from Structure to Human-mediated Process) and the Primary Predictor (the strong significant influence of Perceived Usefulness on Satisfaction, indicated by the bold arrow). By integrating these perspectives, the model provides a holistic lens to explain the ‘Low-Tech, High-Touch’ paradox within the digital health governance of rural Thailand.

## 3. Methodology

### 3.1. The Rongkham Home Ward Model

The ‘Home Ward’ intervention at Rongkham Hospital is a community-based telemedicine system designed for patients with stable chronic conditions (e.g., hypertension, diabetes) or those in post-acute recovery who meet specific safety criteria, such as having a capable caregiver and stable vital signs.

The intervention utilizes a ‘Low-Tech’ infrastructure, primarily leveraging the LINE application—a ubiquitous messaging platform in Thailand—to facilitate daily communication between patients and the multidisciplinary team (MDT). The process is driven by Village Health Volunteers (VHVs), who act as the primary human intermediaries. VHVs visit patients’ homes to collect vital signs (blood pressure, oxygen saturation, and temperature) using portable medical devices and upload the data via a dedicated LINE group. The MDT, based at the hospital, monitors this ‘digital ward’ in real-time, providing tele-consultations and adjusting treatments as necessary. This model shifts the site of care from the hospital bed to the patient’s home, aiming to reduce hospital congestion while maintaining systemic resilience through local social networks.

### 3.2. Research Design

To comprehensively evaluate the efficacy and sustainability of the Home Ward model in a resource-limited setting, this study employed a Convergent Mixed-Methods Design [5]. This approach involved the concurrent collection and analysis of both quantitative and qualitative data to provide a holistic understanding of the research problem.

The rationale for adopting this design was twofold. First, the quantitative strand utilized a cross-sectional survey to measure patient satisfaction and identify its key determinants based on the Technology Acceptance Model (TAM) and the Donabedian Model. This strand aimed to statistically test the relationships between system quality, monitoring processes, and perceived usefulness.

Second, the qualitative strand employed in-depth interviews with two distinct groups: (1) patients and caregivers, to explore their lived experiences and validate the quantitative findings; and (2) the multidisciplinary healthcare team, to uncover the operational mechanisms, specifically the “Human-in-the-Loop” and Service Co-production strategies used to overcome technological limitations.

By integrating these two datasets, the study seeks to triangulate the findings, allowing the high satisfaction scores reported by patients to be contextualized against the systemic resilience and invisible workload managed by healthcare providers. The study was conducted as a single-site case study at Rongkham Hospital, a 30-bed community hospital in Kalasin Province, Thailand, which serves as a representative model for rural healthcare facilities facing similar resource constraints.

### 3.3. Sampling and Participants

This study employed a Convergent Mixed-Methods Design, necessitating a strategic approach to sampling for both strands [5].

For the quantitative strand, a Purposive Census Sampling (*N* = 51) was utilized, encompassing the entire active population of the ‘Home Ward’ pilot program at Rongkham Hospital. This approach was chosen to ensure a complete representation of all patients receiving digital healthcare during the study period.

For the qualitative strand, Maximum Variation Sampling (*n* = 12) was applied to select key informants, including patients, caregivers, and multidisciplinary team (MDT) members. This strategy allowed for the capture of diverse perspectives—ranging from high-frequency users to those with limited digital engagement—thereby enhancing the depth and breadth of the thematic analysis.

#### 3.3.1. Quantitative Strand (Patients and Caregivers)

The study employed a purposive census sampling strategy. The participants (*N* = 51) comprised the entire cohort of patients and caregivers who were actively enrolled in the Rongkham Home Ward pilot program during the study period. Because this was a census of the specific pilot population rather than a sample of a general population, the data captures the full experience of the current intervention.

To address potential concerns regarding the sample size (N = 51), a post hoc power analysis was conducted using G*Power software (Version 3.1.9.6 (for Mac), Heinrich Heine University Düsseldorf (Department of Experimental Psychology), Düsseldorf, Germany). Given the observed large effect size (f^2^ = 2.05), and an alpha of 0.05, the calculated statistical power (1-β) was 0.99, significantly exceeding the conventional threshold of 0.80. This indicates that the study is sufficiently powered to detect statistically significant predictors within the regression model. Furthermore, to mitigate the risk of overfitting, we monitored the Variance Inflation Factor (VIF), which remained between 1.145 and 1.232, confirming the absence of multicollinearity and ensuring the stability of the inferential results.

The participants for the quantitative survey consisted of patients and their primary caregivers who were enrolled in the Home Ward service at Rongkham Hospital. To ensure the data accurately reflected the user experience of this specific service model, purposive sampling was employed based on the following inclusion criteria:

1. Diagnosed with one of the seven eligible conditions for home-based care (e.g., Diabetes Mellitus type 2, Hypertension, Urinary Tract Infection).
2. Accepted into the Home Ward system under the supervision of the multidisciplinary team.
3. Capable of communicating and providing informed consent (or represented by a primary caregiver).

A total of 51 participants (N = 51) completed the survey. While this sample size is reflective of the patient volume in a specialized pilot program at a 30 bed community hospital, it represents a substantial proportion of the active service users during the data collection period. This constitutes a comprehensive sampling of the target population, providing high ecological validity for this specific case study.

It is important to note a potential selection bias inherent in the study design. Participants were selected based on clinical stability and the availability of a caregiver, which suggests a certain level of baseline acceptance of the telemedicine model. Consequently, the high satisfaction ratings should be understood as reflective of a population that met these specific readiness criteria.

#### 3.3.2. Qualitative Strand (Key Informants)

To gain deeper insights into the systemic operations and user experiences, a sub-set of participants was selected for in-depth interviews using a maximum variation sampling strategy. This approach ensured diverse perspectives were captured from two key groups:

Group 1: Service Users (*n* = 5): Comprising patients and caregivers selected to represent different disease types, age groups, and levels of technological literacy. This diversity allowed the study to explore varying degrees of “Perceived Usefulness” and adaptation to the monitoring tools.

Group 2: Service Providers (*n* = 7): Comprising key members of the multidisciplinary team, including physicians, nurses, pharmacists, and Village Health Volunteers (VHVs). These informants were selected based on their direct involvement in the Home Ward operations to provide insights into the “Human-in-the-Loop” mechanisms and service co-production efforts.

Data collection for the qualitative strand continued until data saturation was reached, where no new themes or significant information emerged from subsequent interviews.

### 3.4. Instruments and Measures

To align with the convergent mixed-methods design, two distinct instruments were developed and validated for this study: a structured questionnaire for quantitative measurement and a semi-structured interview guide for qualitative inquiry.

#### 3.4.1. The Quantitative Instrument (Structured Questionnaire)

The quantitative survey was developed by adapting validated scales to fit the rural telemedicine context. Content validity was verified by three experts with an Index of Item-Objective Congruence (IOC) of 0.60–1.00. The quantitative instrument’s reliability was rigorously tested. The analysis yielded an overall Cronbach’s Alpha of 0.843, signifying high internal consistency. Furthermore, each sub-construct was evaluated independently, with all coefficients exceeding the 0.830 threshold. These values confirm that the survey items consistently measure the intended theoretical constructs within the rural Thai context.

The questionnaire was developed based on the Technology Acceptance Model (TAM) and the Donabedian Model to measure the key constructs of the conceptual framework. It consisted of five sections.

Part 1: Demographic Information: Queries regarding age, gender, income, diagnosis, and distance from the hospital.

Part 2: Perceived Usefulness (TAM): Items measuring the patients’ perception of the system’s utility, focusing on convenience, cost-saving, and accessibility compared to traditional hospital admission.

Part 3: System and Process Quality (Donabedian): Items assessing the “Structure” (readiness of medical equipment, telecommunication stability) and “Process” (effectiveness of symptom monitoring, responsiveness of the healthcare team).

Part 4: Patient Satisfaction (Outcome): Items evaluating overall satisfaction with the service delivery, communication channels, and clinical care received at home.

Part 5: Open-ended Suggestions: Allowing participants to report specific problems and recommendations.

All items in Parts 2–4 were measured using a 5-point Likert scale, ranging from 1 (Strongly Disagree/Least Satisfaction) to 5 (Strongly Agree/Highest Satisfaction).

Validity and Reliability: The content validity of the questionnaire was assessed using the Index of Item-Objective Congruence (IOC) by a panel of experts to ensure the questions accurately represented the intended theoretical constructs.

#### 3.4.2. The Qualitative Instrument (Semi-Structured Interview Guide)

To complement the survey data, a semi-structured interview guide was designed to elicit in-depth narratives regarding the “Human-in-the-Loop” aspect and service co-production. The guide was tailored for two groups:

For Patients/Caregivers: Focused on their lived experiences, feelings of safety vs. anxiety, and the tangible benefits of staying at home.

For the Multidisciplinary Team: Focused on operational challenges, workload management, resource allocation, and the mechanisms of coordinating with community health volunteers (VHVs).

### 3.5. Data Collection Procedures

Data collection was conducted over a two-month period from April 2025 to May 2025, following approval from the Human Research Ethics Committee of Rongkham Hospital (Certificate No. 190-062/2568). To ensure the integrity of the convergent design, quantitative and qualitative data were collected concurrently using the following procedures:

#### 3.5.1. Quantitative Data Collection (Survey)

Recruitment: The research team approached eligible patients and caregivers enrolled in the Home Ward system. To minimize potential coercion, it was explicitly stated that participation was voluntary and would not affect their current or future medical treatment.

Administration: For literate participants, the questionnaires were self-administered. For elderly participants or those with visual impairments, trained research assistants utilized a structured interview technique, reading questions aloud and recording responses verbatim to ensure data integrity.

Quality Control: All completed questionnaires were immediately reviewed for completeness. Incomplete forms were addressed on-site by clarifying missing items with the respondents to minimize data attrition.

To mitigate social desirability bias and socio-cultural nuances (e.g., the concept of “Greng-jai”), research assistants followed a strict ‘Neutral Protocol’. This included:

1. Environment: Conducting interviews in a private, non-clinical setting to reduce the perceived power imbalance between providers and patients.
2. Anonymity Assurance: Reiterating that honest feedback would remain confidential and have no impact on healthcare services.
3. Investigator Independence: Utilizing research assistants who were independent of the clinical Multidisciplinary Team (MDT) to ensure participants felt comfortable providing authentic, critical feedback without fear of causing discomfort to their primary care providers.

#### 3.5.2. Qualitative Data Collection (In-Depth Interviews)

Preparation: Key informants were identified and approached based on the maximum variation sampling strategy. Appointments were scheduled at the participants’ convenience, either at their homes (for patients) or private consultation rooms (for staff) to ensure privacy.

Participants for the qualitative strand were selected via purposive sampling to ensure a diversity of perspectives. As detailed in Table 1, this included six service users (*n* = 5) who had completed the Home Ward program and seven healthcare providers (*n* = 7), comprising doctors, nurses, and pharmacists involved in the implementation.

Interview Process: Semi-structured interviews were conducted face-to-face by the principal researcher to establish rapport. The sessions lasted approximately 30–45 min.

Recording: With written consent, all interviews were audio-recorded. Field notes were also taken to capture non-verbal cues and environmental contexts, which are crucial for interpreting the “Human-in-the-Loop” dynamics.

### 3.6. Data Analysis

Data analysis was conducted in two distinct phases corresponding to the convergent design, followed by an integration phase.

#### 3.6.1. Quantitative Data Analysis

Statistical analysis was performed using IBM SPSS Statistics for Macintosh (Version 29.0, IBM Corp, Armonk, NY, USA) for Windows. The analysis proceeded in two steps:

Descriptive Statistics: Frequency, percentage, mean ($\bar{x}$), and standard deviation (S.D.) were calculated to describe the demographic characteristics of the patients and caregivers, as well as their levels of satisfaction and perception of the system.

Inferential Statistics: Multiple Linear Regression (MLR) analysis was employed to test the conceptual framework. Specifically, it examined the influence of Perceived Usefulness (TAM), Effectiveness of Symptom Monitoring (Process), and Overall System Quality (Structure) on Patient Satisfaction (Outcome). Prior to analysis, all assumptions of regression, including normality, linearity, and multicollinearity (VIF values), were verified to ensure the robustness of the model (R^2^ = 0.673).

#### 3.6.2. Qualitative Data Analysis

The qualitative data were analyzed using Thematic Analysis following a systematic two-cycle coding process. In the first cycle, initial coding was conducted to identify emergent labels from the raw interview transcripts. In the second cycle, focused coding was employed to categorize these labels into overarching themes and sub-themes based on the ‘Human-in-the-Loop’ and ‘Systemic Resilience’ frameworks.

To ensure rigor and trustworthiness, the research team utilized manual systematic indexing to manage the data, allowing for close engagement with the Thai cultural and linguistic nuances of the rural context. Inter-coder consensus was achieved through a series of debriefing sessions where the primary researcher and the corresponding author independently reviewed the codes and reconciled discrepancies. Furthermore, member checking was performed by sharing the summarized findings with selected participants to verify accuracy, and data triangulation was achieved by comparing insights from both service users and healthcare providers.

#### 3.6.3. Data Integration

The quantitative and qualitative results were integrated during the interpretation phase using a side-by-side comparison approach. The statistical findings (e.g., high satisfaction scores) were cross-verified with qualitative narratives to identify areas of convergence (confirmation of results) or expansion (where qualitative data explained why specific statistical relationships existed).

### 3.7. Ethical Considerations

This study was conducted in strict adherence to the ethical principles regarding human experimentation as outlined in the Declaration of Helsinki. The research protocol was reviewed and approved by the Human Research Ethics Committee of Rongkham Hospital (Certificate of Approval No. 190-062/2568, dated 12 March 2025).

Prior to data collection, all participants—including patients, caregivers, and healthcare providers—were fully informed about the study’s objectives, procedures, potential risks, and benefits. Informed consent was obtained from all participants. For elderly patients or those with limited literacy, the consent process was facilitated by reading the information sheet aloud and obtaining verbal assent in the presence of a witness, alongside written consent from their legal representatives where applicable.

To ensure confidentiality and anonymity, all personal identifiers were removed from the dataset. Participants were assigned unique code numbers, and data were stored in password-protected files accessible only to the principal investigator. Participation was entirely voluntary; participants were explicitly assured that they could withdraw from the study at any time without providing a reason and without any repercussion on their current or future medical care at Rongkham Hospital.

## 4. Results

### 4.1. Demographic Characteristics of the Participants

**Figure 2:**
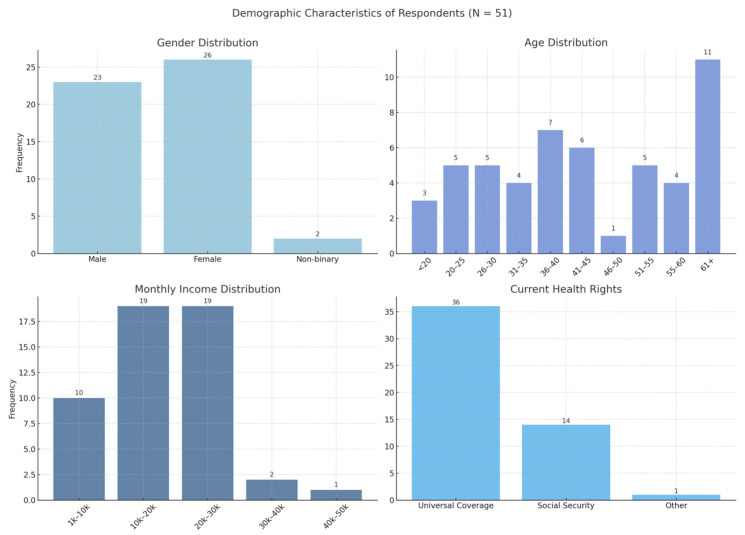
Demographic Characteristics of Respondents.

The quantitative strand of this study involved 51 participants, comprising patients enrolled in the Home Ward service and their primary caregivers. The demographic profile of the respondents and patient service Experience is summarized in Figure 2.

The analysis revealed a relatively balanced gender distribution, with females constituting a slight majority (51.0%) compared to males (45.1%). Regarding age, the participants represented a wide spectrum of age groups, ranging from under 20 years to over 60 years. As illustrated in Figure 2, the largest age group was the elderly population aged 61 years and above (21.6%), reflecting the demographic that typically requires continuous home-based care.

Economically, the majority of participants fell into the lower-to-middle income bracket. Specifically, 74.6% of respondents reported a monthly income between 10,000 and 30,000 THB, while 19.6% earned less than 10,000 THB, highlighting the resource-constrained nature of the community. In terms of healthcare coverage, the vast majority relied on the Universal Coverage Scheme (Gold Card) (70.6%), followed by the Social Security Scheme (27.5%). This underscores the reliance of the target population on public welfare systems for healthcare access.

### 4.2. Clinical Characteristics and Service Accessibility

**Figure 3:**
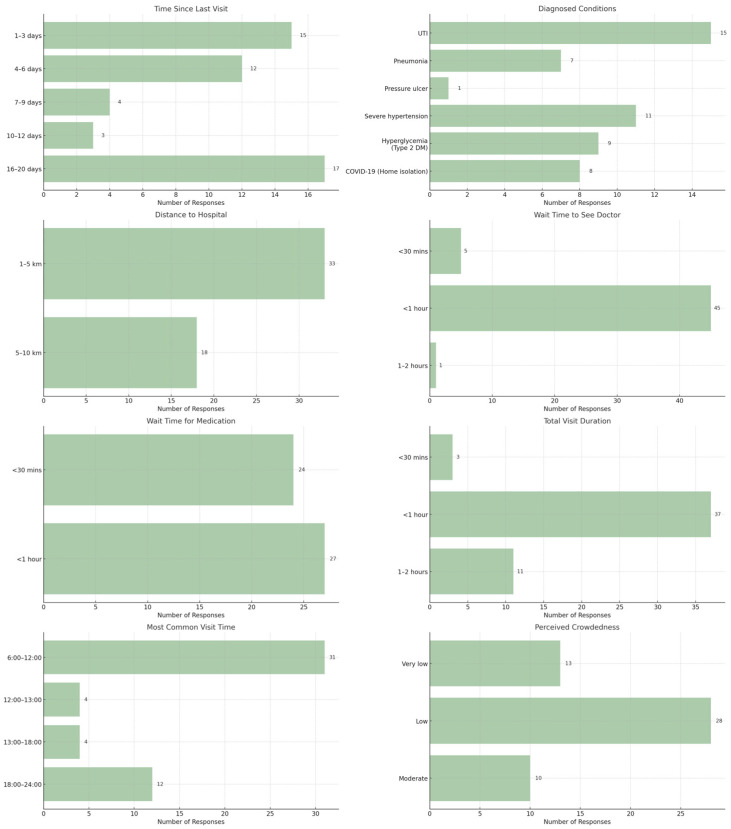
Summary of Patient Service Experience.

Beyond demographic profiles, the study analyzed the clinical background and service experience of the participants (N = 51). As illustrated in Figure 3, the most prevalent diagnosis among Home Ward patients was Urinary Tract Infection (UTI) (*n* = 15), followed by Hypertension (*n* = 11) and Hyperglycemia (*n* = 9). Data regarding the duration of previous hospitalizations showed a bimodal distribution, with patients predominantly staying either for a short duration (1–3 days) or a prolonged period (16–20 days), reflecting the varied acuity of cases managed at home. Regarding service accessibility, the majority of patients resided within a 1–5 km radius of Rongkham Hospital (*n* = 33), facilitating convenient access to care. Service efficiency was a notable strength of the hospital; a significant proportion of patients reported waiting times of less than one hour to see a physician (*n* = 45) and to receive medication (*n* = 27). Furthermore, the total service completion time was predominantly under one hour (*n* = 37). Consistent with these efficiency metrics, patients perceived the level of hospital congestion as low to least congested, which aligns with the high satisfaction scores reported in the subsequent analysis.

### 4.3. Quantitative Findings

#### 4.3.1. Descriptive Analysis of Key Study Variables

**Figure 4:**
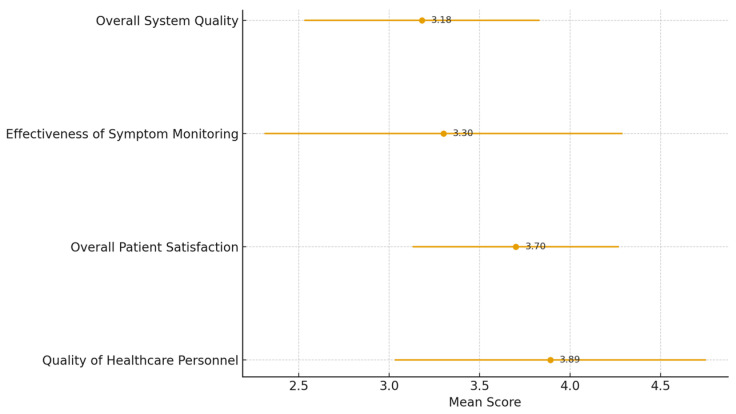
Descriptive Statistics of Key Study Variables (N = 51).

The quantitative analysis is presented in two distinct stages. First, descriptive statistics (Figure 4) were employed to evaluate the general perceptions and satisfaction levels of the participants across all dimensions. Second, Multiple Linear Regression (MLR) (Table 2) was utilized as an inferential tool to identify which specific constructs significantly predict overall satisfaction. While the descriptive results provide a baseline of participant sentiment, the regression model isolates the causal strength of each predictor, accounting for potential confounding between variables.

Prior to examining the causal relationships, descriptive statistics were analyzed to assess the participants’ levels of perception regarding the Home Ward service. As shown in Table 2, the results reveal a distinct contrast between the “human” and “technological” elements of the service. Participants rated the Quality of Healthcare Personnel highest ($\bar{x}$ = 3.89, S.D. = 0.86), reflecting strong confidence in the multidisciplinary team and volunteer support. In contrast, Overall System Quality received the lowest rating ($\bar{x}$ = 3.18, S.D. = 0.65), indicating potential limitations in the structural or technological infrastructure. Despite these systemic constraints, the Overall Patient Satisfaction remained at a high level ($\bar{x}$ = 3.70, S.D. = 0.57), suggesting that the dedication of the personnel effectively compensates for the system’s shortcomings.

#### 4.3.2. Determinants of Patient Satisfaction in Home Ward Services

The Multiple Linear Regression (MLR) model demonstrated high statistical stability. The Variance Inflation Factor (VIF) values ranged from 1.145 to 1.232, well below the conservative threshold of 5.0, confirming the absence of multicollinearity. Furthermore, despite the modest sample size (N = 51), the post hoc power analysis yielded a power of 0.99 for the observed effect size (f^2^ = 2.05), validating that the model is sufficiently powered to detect the reported predictors.

A multiple linear regression analysis was conducted to predict overall patient satisfaction based on three key predictors: (1) Perceived Usefulness of the Home Ward system, (2) Effectiveness of Symptom Monitoring, and (3) Overall System Quality. The results indicated that the regression model was statistically significant (F(3, 47) = 32.289, *p* < 0.001) and achieved a high level of prediction accuracy, explaining approximately 67.3% of the variance in patient satisfaction (R^2^ = 0.673, Adjusted R^2^ = 0.652). Table 2 presents the regression coefficients. The analysis revealed that Perceived Usefulness (β = 0.589, *p* < 0.001) and Effectiveness of Symptom Monitoring (β = 0.317, *p* < 0.001) were significant positive predictors of patient satisfaction. However, Overall System Quality did not significantly predict satisfaction in this model (β = 0.142, *p* > 0.05). highlighting the “Low-Tech, High-Touch” nature of the service.

**Table 2:** Multiple Linear Regression Predicting Overall Patient Satisfaction with the Home Ward System.

Predictor Variable	B	Std. Error	β (Beta)	t	Sig.	VIF
(Constant)	1.028	0.295	–	3.488	0.001 **	-
Perceived Usefulness of Home Ward	0.428	0.067	0.589	6.368	0.000 ***	1.232
Effectiveness of Symptom Monitoring	0.182	0.051	0.317	3.555	0.000 ***	1.145
Overall System Quality	0.125	0.080	0.142	1.573	0.123	1.181

Note: 1. Significance levels: ** *p* < 0.01; *** *p* < 0.001. Predictors were entered using the Enter method. 2. R = 0.821, R^2^ = 0.673, Adjusted R^2^ = 0.652, Std. Error of Estimate = 0.33447 Dependent Variable: Overall Patient Satisfaction.

### 4.4. Qualitative Findings: The Human Dimension of Home Ward

The qualitative strand was designed to deconstruct the ‘invisible workload’—the latent, undocumented labor that serves as a critical bridge between constrained digital infrastructure and superior patient outcomes. While the quantitative findings demonstrated high satisfaction despite moderate system quality, the qualitative inquiry illuminates the socio-technical mechanisms that drive this success. Through an interpretive analysis of in-depth interviews with service users (*n* = 5) and the multidisciplinary team (*n* = 7), the study moved beyond surface-level reporting to explore how systemic resilience is co-produced. This process identified three emergent themes that characterize the ‘High-Touch’ interventions required to sustain rural telemedicine.”

#### 4.4.1. Theme 1: “Pragmatic Utility” as a Survival and Cultural Strategy

Consistent with the quantitative finding that Perceived Usefulness (PU) is the most potent predictor of satisfaction (β = 0.589, *p* < 0.001), patient narratives underscored that the value of the Home Ward lies not in the sophistication of the digital platform, but in its ability to mitigate the systemic vulnerabilities of rural life.

- **Economic Resilience and Livelihood Preservation**

Patients and caregivers did not view the Home Ward merely as a convenience; they framed it as a critical survival strategy within a fragile agrarian economy. The utility of the system is rooted in its capacity to prevent “catastrophic health expenditure” by eliminating indirect costs such as travel, hospital-stay food, and, most importantly, the opportunity cost of labor.

“Staying at home saves us everything. No travel costs, no need for my children to take leave from work to guard me at the hospital. It’s not just about comfort; it’s about survival for the family.” (Patient #SU03, Hypertension)

- **Psychological Safety and the Restoration of Human Agency**

Beyond economic relief, the home environment provides “psychological safety”—a state where the patient is no longer a passive recipient of care but an active agent in their own recovery. The Home Ward allows for a seamless integration of health management into the rhythms of rural life, preserving the patient’s identity and human dignity.

“At the hospital, I feel pressured. I worry about the farm, the house. Here, I take my medicine, measure my blood pressure, and then I can feed my chickens. I feel like a person, not just a patient.” (Patient #SU05, Diabetes)

- **Sustaining Filial Piety (Family Duty) as a Socio-Technical Mediator**

A significant interpretive layer identified in this study is the role of technology in sustaining “Filial Piety” (Family Duty). In rural Thai society, caring for aging parents is a non-negotiable moral imperative. The Home Ward acts as a socio-technical mediator that resolves the tension between modern economic demands and traditional moral obligations. This cultural alignment is a fundamental, albeit invisible, driver of the system’s high satisfaction scores.

“I want to be a good daughter and take care of my mother myself, but the hospital stays made it impossible to keep my job. I used to have to choose between my duty to her and my duty to my employer. This system bridges that gap. It gives me the tools to watch over her while I continue to support the family economically.” (#SU04, Caregiver/Relative & VHV)

#### 4.4.2. Theme 2: The “Human-in-the-Loop” as the Systemic Backbone

The interviews with the multidisciplinary team (MDT) illuminated a critical finding: the resilience of the Home Ward system depends less on the stability of the digital platform and more on the “Human-in-the-Loop” (HITL) mechanism. This human agency acts as a compensatory layer that bridges the gaps in digital infrastructure.

- **The “Digital Bridge”: Mediating the Technological Divide**

Providers emphasized that in a rural aging society, digital literacy is a significant barrier. The functionality of the telemedicine link is, therefore, sustained by Village Health Volunteers (VHVs) and nursing staff who serve as “digital bridges.” They translate raw health data into digital format, ensuring that technological constraints do not lead to social exclusion.

“The technology itself is basic—primarily the LINE application—but even that can be an insurmountable barrier for the elderly. The real engine of this system is the VHVs. They visit the homes, take the measurements, and capture the photos for us. They are the ‘physical’ link that prevents the ‘digital’ link from breaking. Without this human mediation, the system would be inaccessible to those who need it most.” (#HP03, Registered Nurse, Operational Coordinator)

- **Service Co-production: Interdependence in Resource-Constrained Settings**

The narratives confirmed that care delivery is not a top-down medical service but a co-produced effort. In this model, physicians act as strategists, nurses as operational managers, and families/VHVs as the “eyes and ears” on the ground. This high level of interdependence compensates for the typical resource constraints of a community hospital, creating a “High-Touch” environment within a “Low-Tech” framework.

“We don’t just treat the patient; we manage a network. As a doctor, I rely entirely on the information sent by the caregivers and Village Health Volunteers (VHVs). We are co-producing health. My clinical decision is only as good as the ‘eyes and ears’ we have in the village. This interdependence is what makes the system resilient—it’s about trust, not just tools.” (Physician, HP01, Policy Oversight)

- **The Invisible Workload: Emotional and Operational Labor**

Beyond clinical duties, the MDT highlighted the “invisible workload” required to maintain system quality. This includes the emotional labor of constant communication and the operational agility needed to troubleshoot technical and logistical issues in real-time.

“People see the dashboard and think it’s automated. It isn’t. Every data point on that screen represents a phone call, a home visit, or a chat message to encourage the patient. This ‘invisible labor’ is what keeps the satisfaction levels high, even when the internet signal or the devices fail us.” (#HP04, Senior Nurse Manager).

#### 4.4.3. Theme 3: Systemic Resilience Amidst Operational Burdens

Despite the clinical and logistical success of the Home Ward, the provider narratives uncovered a substantial layer of “invisible workload”—a latent labor force that the quantitative data, focused on patient satisfaction, could not fully capture. This theme highlights that the system’s resilience is built upon the personal dedication of the staff rather than an optimized organizational structure.

- **The Dual-Burden of Care: Navigating Structural Constraints**

The providers expressed significant concerns regarding the dual-burden of care. Most staff members are required to manage their primary responsibilities in the in-patient department (IPD) or outpatient clinics while simultaneously overseeing the Home Ward. Without additional manpower, the high satisfaction reported by patients (Figure 4) is often co-produced through the extra-role behaviors and emotional labor of the medical team.

“We are happy the patients are happy. But the reality is we are working double. We do the ward rounds in the morning and then answer LINE messages for Home Ward patients until late at night. It’s sustainable only because we care about our community.” (Physician #01)

- **Sustainability vs. Personal Dedication**

The narratives suggested a precarious balance between systemic sustainability and individual sacrifice. While the “High-Touch” nature of the service drives patient trust, the providers warned that relying on the “altruism of the community” might lead to burnout if the organizational infrastructure is not formalised.

“This system is sustainable only because we are a community hospital and we care about our neighbors. But from a management perspective, it’s taxing. We are bridging the gaps of a ‘Low-Tech’ system with ‘High-Intensity’ human effort. To scale this up, we need more than just dedicated people; we need a system that recognizes this digital labor as part of our core duty.” (#HP04, Senior Nurse Manager)

- **The Resilience Paradox**

The qualitative evidence points to a “Resilience Paradox”: the very factor that makes the Home Ward successful (the intensive human-in-the-loop mediation) is also its greatest vulnerability. The systemic resilience observed is not a product of technical automation, but a result of socio-technical adaptation where humans compensate for technological and resource limitations.

“People see the high satisfaction scores and think the technology is doing the work. They don’t see the nurses troubleshooting the devices at 8 PM or the pharmacists carefully re-explaining medication through a chat app. That ‘invisible labor’ is the true backbone of the system’s resilience.” (#HP01, Physician, Policy Oversight)

### 4.5. Mixed-Methods Integration: Meta-Inferences on Systemic Resilience

To provide a holistic understanding of the Home Ward system, the quantitative and qualitative findings were integrated into a Joint Display (Table 3). This integration moves beyond simple convergence to generate Meta-inferences—overarching conclusions that explain the causal mechanisms behind the observed success of the program.

## 5. Discussion

The findings of this study reveal a compelling “Socio-Technical Paradox”: while the technical infrastructure and system quality of the Home Ward received only moderate ratings ($\bar{x}$ = 3.52), overall patient satisfaction remained high ($\bar{x}$ = 3.70). This indicates that in rural telemedicine, service success is not a function of technological sophistication, but a result of Pragmatic Utility and Socio-Technical Mediation.

### 5.1. Mitigating the Medical Poverty Trap Through Pragmatic Utility

The quantitative finding that Perceived Usefulness (PU) was the most potent predictor of satisfaction (β = 0.589, *p* < 0.001), while System Quality had no significant impact, underscores that technology adoption in LMICs is driven by its capacity to solve fundamental socio-economic challenges. Our qualitative data clarifies that “utility” in the rural Thai context is synonymous with Financial Risk Protection (FRP).

The Home Ward functions as a vital safeguard against the “medical poverty trap”—a cycle where health shocks lead to significant out-of-pocket (OOP) expenses and subsequent impoverishment [36,37]. In many LMICs, health-seeking behavior is often constrained by catastrophic health expenditure, forcing households to adopt detrimental coping strategies such as borrowing or selling productive assets [38]. By relocating care to the domestic sphere, the Rongkham model effectively eliminates travel costs and the opportunity cost of labor, preventing vulnerable agrarian households from falling into health-induced debt [39]. This confirms that increased service coverage can mitigate poverty gaps if the technology effectively addresses the user’s economic pain points [40] [41].

### 5.2. From Structure to Process: The “Human-in-the-Loop” as a Compensatory Layer

Applying the Donabedian Model, this study demonstrates a strategic shift from Structure to Process. While structural deficiencies in digital infrastructure existed, the Process—operationalized through the Effectiveness of Symptom Monitoring—remained a significant predictor of satisfaction (β = 0.317, *p* < 0.001). This underscores a compensatory mechanism where the agency of healthcare personnel and the strength of the therapeutic alliance mitigate structural limitations.

Unlike Hospital-at-Home (HaH) models in advanced economies such as Singapore or the United States, which often grapple with technological literacy and communication barriers [42,43], the Rongkham model leverages deep-seated Social Capital. Village Health Volunteers (VHVs) serve as “digital bridges,” ensuring system inclusivity for populations with limited technological proficiency [44]. This ‘High-Touch, Low-Tech’ approach effectively compensates for constrained hospital infrastructure, echoing the findings of Tan et al. (2025) [45] that HaH models can maintain high safety and satisfaction while reducing healthcare expenditures. Furthermore, these findings extend the discourse on Street-Level Bureaucracy; frontline healthcare workers effectively “re-make” digital policy on the ground, exercising discretionary agency to achieve health equity despite the digital divide.

### 5.3. The Resilience–Exploitation Nexus: Ethical Dimensions of Altruism

A critical ethical tension identified in this study is the Resilience–Exploitation Nexus. While the ‘Rongkham Model’ demonstrates high systemic resilience through service co-production, this resilience is largely ‘borrowed’ from the personal time and dedication of the multidisciplinary team. As noted by Rothman et al. (2024) [46], the success of Hospital-at-Home (HaH) models often hinges on providers exceeding their contractual duties—a phenomenon [46] we term ‘Altruistic Subsidy.’ When digital health policies rely on the ‘invisible labor’ of staff to compensate for structural deficiencies, they risk transitioning from co-production to informal exploitation. This is particularly evident in our qualitative findings, where staff manage 24/7 communications without additional manpower or compensation. Consistent with O’Donnell (2024) [47], we argue that for such models to be sustainable in LMICs, the ‘human-in-the-loop’ must be formalized. The policy must move beyond praising staff dedication to implementing supply-side interventions—such as digital labor recognition and workload recalibration—to ensure that systemic resilience does not result in provider burnout [42]. Ultimately, when individual altruism is treated as the primary engine of a system without official formalization, it raises significant ethical questions regarding the long-term viability of the policy itself.

### 5.4. The Sustainability Paradox: Co-Production and Invisible Labor

The resilience of this model relies on Service Co-production, where care is a shared responsibility between the state, the community, and the family. However, our meta-inference uncovers a Sustainability Paradox. While patients perceive reduced burdens, the system’s resilience is “borrowed” from the invisible workload of providers—extra-role behaviors such as after-hours monitoring and double-charting (HP02, HP04).

This reliance on individual altruism poses a challenge for long-term viability. As noted by Rothman et al. (2024) [46] and O’Donnell (2024) [47], the success of HaH depends on addressing supply-side interventions and ensuring adequate support for both patients and providers. Without proactive policy intervention to formalize this digital labor, the model remains at risk of collapse due to workforce exhaustion. To scale this model effectively, a multifaceted approach is essential—one that includes expanding health coverage and implementing social protection measures that officially recognize and compensate for the latent labor required to sustain rural telehealth [48].

## 6. Limitations and Future Directions

### 6.1. Sample Size and Statistical Generalizability

While the sample size (N = 51) is relatively small and might typically limit broad statistical generalizability, its impact in this study is mitigated by the comprehensive census approach, which captures the entire active population of the Home Ward pilot at the study site. This provides high ecological validity, ensuring that the findings accurately reflect the lived realities of the specific rural pilot context.

The potential risk of model overfitting was strictly monitored using the Variance Inflation Factor (VIF), which ranged from 1.145 to 1.232, confirming the absence of multicollinearity and ensuring the stability of the regression coefficients. Furthermore, the high statistical power (0.99) achieved despite the modest *N* demonstrates the exceptional strength of the identified predictors, such as Perceived Usefulness, in this setting.

However, readers should exercise caution when extrapolating these findings to larger, urban environments. The “Human-in-the-Loop” mechanism identified here relies heavily on local social capital and the cohesive VHV network, which may differ significantly in more transient or highly automated urban healthcare settings.

### 6.2. Cultural Nuances and Social Desirability Bias

The cultural nuance of ‘Greng-jai’—the reluctance to criticize figures of authority or benefactors—may have influenced the authenticity of responses, particularly among elderly participants who hold deep respect for healthcare providers. While the study utilized triangulation through a mixed-methods approach to verify consistency between quantitative ratings and qualitative narratives, the possibility of inflated satisfaction scores remains a limitation.

Furthermore, a potential selection bias must be acknowledged. As this was a pilot study with specific “digital readiness” and clinical stability criteria for enrollment, the participants may possess a higher “baseline acceptance” of telemedicine compared to the general population. Consequently, the high satisfaction ratings should be interpreted as reflective of a population that already possessed a degree of openness to technological intervention. Future longitudinal studies should include more diverse patient profiles, including those with lower digital literacy, to further test the model’s systemic resilience.

### 6.3. Focus on Perceptual vs. Clinical Outcomes

As a cross-sectional study, this research captures a snapshot of service perceptions and process satisfaction within the Donabedian framework. It does not measure long-term clinical outcomes, such as recovery rates, long-term mortality, or the prevention of chronic complications. While satisfaction is a critical precursor to system adoption, longitudinal research is essential to evaluate whether the “Human-in-the-Loop” model translates into sustained clinical efficacy over time.

### 6.4. Contextual Transferability

The findings possess high ecological validity for rural community hospitals that operate within strong community networks. However, the transferability of the “Rongkham Model” to urban settings—where social capital and the VHV network may be less cohesive—is limited. Future studies should explore how the “Human-in-the-Loop” mechanism can be adapted to urban environments where the “digital bridge” provided by volunteers might be absent.

## 7. Conclusions and Policy Implications

### 7.1. Theoretical and Practical Contributions

The findings reveal a compelling paradox where moderate ratings for system infrastructure coexist with high levels of patient satisfaction. This success is underpinned by the integration of accessible, appropriate technology with strong social capital, specifically the collaboration between multidisciplinary healthcare teams and community networks. The study confirms that in the context of rural Thailand, the “Human-in-the-Loop” mechanism—where Village Health Volunteers (VHVs) and healthcare providers act as “digital bridges”—effectively compensates for the digital divide, ensuring that technological illiteracy does not lead to health exclusion.

Furthermore, the research establishes that the adoption of the Home Ward service is motivated largely by Pragmatic Utility. Patients prioritize tangible economic benefits—such as the mitigation of the medical poverty trap through reduced travel and caregiver opportunity costs—over the sophistication of the digital platform itself. This supports the proposition of a “Low-Tech, High-Touch” model, suggesting that for developing nations, sustainable healthcare solutions must be responsive to the socioeconomic realities of the community.

### 7.2. Governance and Sustainability Recommendations

Crucially, this study contributes to the governance literature by framing healthcare providers as Street-Level Bureaucrats (SLBs). These workers exercise significant discretion to bridge the gap between policy ideals and resource scarcity. However, the study uncovers that the current system’s resilience relies heavily on the “invisible workload” and the extra-role altruism of staff. While these SLBs effectively “remake” telemedicine policy on the ground, this reliance on individual sacrifice poses a significant threat to long-term systemic durability.

Therefore, to transition from a pilot success to a sustainable Decentralized Digital Health Governance framework, policymakers must move beyond technical provision. Sustainable implementation requires:

- Structural Support: Formalizing the digital labor of healthcare workers to prevent burnout.
- Social Protection for Intermediaries: Recognition and compensation for VHVs who act as the system’s “eyes and ears.”
- Resource Allocation: Dedicated staffing for Home Ward operations rather than relying on dual-role burdens.

In conclusion, this study offers a set of human-centric design principles for other Low- and Middle-Income Countries (LMICs). Rather than a universal prescription, the Rongkham Model serves as an illustrative framework for similar rural contexts, demonstrating that the heart of sustainable healthcare lies not in the technology itself, but in empowering the human network that sustains it.

## Figures and Tables

**Table 1:** Socio-Demographic Characteristics of Key Informants for Qualitative Inquiry (*N* = 12).

Code	Gender	Age	Role/Position	Department/Diagnosis	Experience/Context
Panel A: Healthcare Providers (*n* = 7)					
HP01	Male	37	Physician	Medical Dept.	Oversees Home Ward policy
HP02	Female	38	Physician	Medical Dept.	Manage clinical decisions
HP03	Female	23	Registered Nurse	In-patient Dept. (IPD)	Operational coordinator
HP04	Female	50	Registered Nurse	In-patient Dept. (IPD)	Senior nurse manager
HP05	Female	56	Registered Nurse	Out-patient Dept. (OPD)	Pre-admission screening
HP06	Female	29	Pharmacist	Pharmacy Dept.	Medication management
HP07	Female	36	Registered Nurse	Emergency Room (ER)	Emergency support
Panel B: Service Users (*n* = 5)					
SU01	Female	53	Patient	Hypertension (HTN)	Self-care capability
SU02	Male	53	Patient	Diabetes (DM)	Severe history (amputation risk)
SU03	Female	25	Caregiver (Daughter)	Pressure Ulcer	Managing bedridden patient
SU04	Female	38	Caregiver (Relative)	Hypertension (HTN)	Also serves as VHV (Village Health Volunteers)
SU05	Female	59	Caregiver (Relative)	Diabetes (DM)	Limited tech literacy

Note. HP = Healthcare Provider; SU = Service User; VHV = Village Health Volunteer. Data regarding SU06 represents the final participant required to reach data saturation (if applicable based on full dataset).

**Table 3:** Joint Display of Integrated Findings and Meta-inferences.

Key Theme	Patient/Caregiver Perspective (Quant & Qual)	Provider/Multidisciplinary Team Perspective (Qual Only)	Integration Outcome
Pragmatic Utility vs. Strategic Efficiency	High Satisfaction: Driven by “Survival Utility”—the mitigation of travel costs and preservation of household labor (SU03).	Operational Stewardship: Focused on managing clinical risk and optimizing bed occupancy in a resource-constrained hospital (HP01).	Synergistic Alignment: While motivations differ (survival vs. efficiency), both groups converge on Perceived Usefulness. The system succeeds because it aligns with the socio-economic survival needs of the poor and the strategic needs of the provider.
Socio-Technical Mediation (Human-in-the-Loop)	Relational Trust: Low concern for technical features; high reliance on the “Digital Bridge” provided by VHVs and nurses (SU04).	Compensatory Labor: Identified “Digital Divide” as a systemic risk, mitigated by human intermediaries acting as the system’s backbone (HP03).	Compensatory Resilience: Meta-inference: Technical infrastructure quality (β = 0.142, *p* > 0.05) is secondary because Human Social Capital proactively fills the functional gaps. The “High-Touch” network effectively compensates for the “Low-Tech” platform.
Workload and Sustainability	Burden Relief: Experienced reduced burden (no travel, comfortable home environment).	Invisible Workload: Experienced increased workload (double charting, 24/7 LINE responses) without extra manpower.	Divergence (Systemic Resilience): Highlights a critical trade-off. High patient satisfaction is currently sustained by the extra effort of providers, raising questions about long-term sustainability.

## Data Availability

The data presented in this study are available on request from the corresponding author due to ethical restrictions regarding participant privacy and confidentiality.
